# The Pleiotropic Effects of Gut Microbiota in Colorectal Cancer Progression: How to Turn Foes into Friends

**DOI:** 10.3390/cancers15030916

**Published:** 2023-02-01

**Authors:** Samuele Tardito, Serena Matis, Roberto Benelli

**Affiliations:** SSD Oncologia Molecolare e Angiogenesi, IRCCS Ospedale Policlinico San Martino, 16132 Genova, Italy

## 1. Introduction

Colorectal Cancer (CRC) is one of most frequent malignant cancers, showing high lethality worldwide [1]. In the past, CRC was more frequently diagnosed in Western countries, but the recent economic development of other areas and spread of occidental habits are increasing CRC incidence in developing countries too [2,3]. These data indicate that environmental elements and lifestyle habits are important in triggering CRC carcinogenesis [4,5]. Red processed meat, saturated fats, and alcohol consumption, along with smoking, obesity, and physical inactivity, are linked to an increased CRC susceptibility [4]. Indeed, many CRC determinants could act as risk factors contributing, with different penetrance, to the overall cancerogenic effect. Nevertheless, CRC carcinogenesis is a very slow process that can manifest over 10 years, depending on the prevalence of genetic and epigenetic alterations. 

Food does not act only directly on gut wellness [6,7]. Indeed, many studies described the importance of dietary habits in the modulation of gut microbiota (GM) composition, which in turn can act as a cofactor in CRC development [8,9]. The GM can be considered as a metabolic organ with a symbiotic affinity to the gut. Many different bacterial species contribute to GM composition, exerting several roles in maintaining the intestinal homeostasis. Healthy commensal bacteria produce essential metabolites from the fermentation of dietary fibers, preserving the GM equilibrium and reducing the infestation of pathogenic strains [9]. Another fundamental activity exerted by GM is the activation of the host immune system [10,11]. 

Bad eating habits, alcohol consumption, and cigarette smoking may favor the development of dysbiosis by the increased proliferation of aggressive bacterial (such as Fusobacterium (F.) nucleatum and Porphyromonas), viral, and fungal species [12]. This would lead to an imbalance in the composition, metabolism, and function of GM, which may precede the onset of CRC and several other gut diseases [13,14]. While everyone’s genetics is important in determining the final equilibrium of a personal GM, once the composition of bacterial flora is established, it is stable over time. Nevertheless, it still shows a relative plasticity when adapting to gut-perturbing factors [15,16]. Only a prolonged diet with junk food could progressively reduce the variety of favorable bacterial species, affecting intestinal homeostasis and leading to dysbiosis and epithelial permeability [17]. In addition to a healthy diet, regular physical exercise [18,19] and meditation [20] can positively affect gut wellness, promoting GM biodiversity and an enrichment in healthy bacteria, actively protecting the intestinal barrier from pathogens.

Alcoholado LS et al. proposed a valuable overview on the role of the GM in the development of CRC, indicating some strategies to prevent tumor onset [21]. In particular, they focused on the importance of the GM composition as a tiebreaker in promoting or contrasting dysbiosis, epithelial inflammation, and CRC. This review indicates bacterial metabolites promoting dysbiosis and how antibiotics reduce the bacterial protective biofilm favoring tissue invasion by pathogenic strains. On the other hand, the authors indicate diet-derived molecules (i.e., fibers and polyunsaturated fatty acids) that, once fermented by bacteria, contribute to maintain a healthy GM biocenosis. Finally, probiotics and bioactive compounds such as quercetin, anthocyanin, tannins, and curcumin are analyzed. These diet components can help to prevent several gut pathologies, are able to actively contrast some phases of tumor life-history, and can decrease the side effects of chemotherapy [21].

## 2. Microbiota and CRC Risk

Anaerobic fermentation operated by the gut microbiota produces different metabolites. Among those derived from undigested dietary components, there are polyamines. It is now evident that a CRC-associated microbiome can be directly involved in the dysregulated metabolism of polyamines [22]. Even if polyamines are fundamental constituents for normal cell growth, the data from a metabolomic screening of CRC tissues [22] revealed that the development of bacteria biofilm lining the gut is dependent on host-enhanced polyamine metabolism. Since polyamines are necessary components for cell membrane synthesis, they could directly contribute to the proliferation of CRC cells [22]. 

Antibiotics are additional risk factors that could indirectly promote colon carcinogenesis, affecting gut microbiota homeostasis [23]. Their ability to suppress many bacterial strains is associated with the decrease in crypt height and heme-induced lipoperoxidation under an enriched red-meat diet [24]. According to nested case–control studies on humans, the pro-oncogenic activity of antibiotics could be linked to either an imbalance of bacterial populations (also favoring fungi outgrowth) or the sudden increment of bacteria after antibiotics withdrawal [25,26]. Importantly, a chronic perturbation of gut microbiota at the end of antibiotics treatment might influence the long-term dysregulation of host immune homeostasis, impacting the immune reaction against CRC [23]. Finally, antibiotic-induced dysbiosis might also decrease the therapeutic efficacy of orally administered anti-cancer drugs, limiting their uptake by enterocytes [27]. 

## 3. Prevention of CRC Carcinogenesis

Epidemiological and clinical studies have found that diet plays a critical role in the induction or prevention of CRC. Since dietary products impact the gut microbiome, their reciprocal influence on CRC is evident. A diet enriched with fibers favorably affects the metabolic activities of the GI tract [28]. Interestingly, dietary fibers also reduce the risk of CRC. This favorable effect is linked not only to the quantity of fibers but also to their density [29]. Although, in the human-based EPIC study, the source of fibers was not apparently relevant [30], some mouse models suggest that short-chain carbohydrates, such as inulin, could be more beneficial [31]. Moreover, a higher intake of dietary fibers could improve patient survival also after CRC diagnosis [32].

The most important effect of dietary fibers is the favorable modulation of microbiota composition. In particular, a high-fiber diet diminished the risk of developing F. nucleatum-positive CRC [33], with F. nucleatum being responsible for increased inflammation and impaired immune reaction, which are both implied in colon carcinogenesis [34]. High fiber diets also increase the presence of butyrate-producing bacteria, which are important to ferment soluble fibers to short chain fatty acids (SCFAs) that, in turn, play a critical role in cancer prevention [35]. Saccharolytic fermentation and butyrogenesis induced by fibers are accompanied by the suppression of secondary bile acid synthesis, which is linked to a reduction in CRC risk [36].

Additionally, the assimilation of polyunsaturated fatty acids (PUFAs), such as omega-3, with diet could be beneficial to reduce CRC risk [37]. Similar to a high fiber intake, supplementation with PUFAs can decrease the presence of pathogenic bacteria and increase the presence of butyrate-producing bacteria. Of note, PUFAs act against the development of CRC also regulating the differentiation and apoptosis of colonocytes [38]. Moreover, they can act on the immune system and modulate CRC-related gene expression, decreasing the risk of high microsatellite instability (MSI) and possibly improving the DNA repair systems [39]. Nevertheless, since PUFAs composition is different in cancer and normal tissue, the metabolism of PUFAs also plays a role in inflammation and in the development of CRC [40].

Probiotics are valuable diet supplements. A regular supplementation with well-defined, healthy bacterial strains can modulate the composition of GM, reducing the inflammation caused by dysbiosis and favoring the restoration of the equilibrium [41]. Many probiotics are able to decrease gut epithelial cell permeability, increase the activity of the host immune system, indirectly activating the phagocytosis of cancer cells and reducing the side effects of chemotherapy [42,43].

Among the classes of phytochemicals that are able to favor eubiosis, there are polyphenols. These aromatic compounds are found at high concentrations in coffee, tea, cocoa, wine, fruits, vegetables, and whole-grain cereals. They can act both directly on host cells and on resident GM, being able to modulate its composition inhibiting the proliferation of many bacterial and viral strains. In CRC, polyphenols could significantly modulate the DNA repair system, decrease metastatic spread, and inhibit tumor neo-angiogenesis [44]. Moreover, polyphenol assumption could increase the anti-cancer potential of some drugs [45,46,47]. 

## 4. Conclusions

The review of Alcoholado et al., starting from the overview of the mechanisms mediating the involvement of GM in the development of CRC, underlies how gut microbiota can be modulated to contrast it. As reported, a regular intake of positive modulators (fibers, PUFAs, and probiotics) can avoid dysbiosis and intestinal inflammation, thus contributing to CRC prevention. Importantly, the plasticity of the microbiome could also impact the host’s environmental fitness in the short or long term, improving or reducing the assimilation of nutrients or toxins [15]. In this perspective, species-specific GM could have co-evolved in parallel with their host.

As the influences of GM and the gut are reciprocal, behaviors able to modify gut motility or food adsorption can strongly perturb microbiome homeostasis. For example, physical activity might change the bacterial composition and counteract dysbiosis in CRC patients [18,19,48]. Moreover, there are some data about meditation-based therapy that, reducing stress, could indirectly act on the health of GM [20,49].

In support of numerous hypotheses, the EPIC perspective trial followed 519,978 healthy human subjects from 10 countries over years [30]. EPIC was able to show a clear, favorable relation between fiber consumption and reduced CRC onset. Large, perspective epidemiologic studies on the healthy human population such as EPIC are usually prohibitive in terms of costs and time, thus alternative approaches are needed. Using GM composition analysis as a surrogate marker of gut wellness, clinical and preclinical studies might be implemented to identify multidisciplinary (diet, exercise, and meditation) approaches able to prevent or delay CRC onset. In the meantime, the education of the general population towards healthier lifestyle habits appears to be the only immediate approach for limiting CRC incidence.
